# Suicidality and epilepsy: A systematic review and meta-analysis

**DOI:** 10.3389/fpsyt.2023.1097516

**Published:** 2023-03-29

**Authors:** Haijiao Wang, Yu Zhang, Ge Tan, Deng Chen, Yaoqi Fu, Ling Liu

**Affiliations:** ^1^Department of Neurology, West China Hospital, Sichuan University, Chengdu, China; ^2^Department of Neurology, The Third Xiangya Hospital, Central South University, Changsha City, China; ^3^Department of Neurology, Chengdu Shangjin Nanfu Hospital, Chengdu, China

**Keywords:** epilepsy, suicidal ideation, suicide attempt, completed suicide, incidence rate

## Abstract

**Background:**

We aimed to evaluate the association between epilepsy and suicidality, including suicidal ideation, attempts and completed suicide.

**Methods:**

We systematically searched PubMed, Embase, Cochrane Online Library, and Clinicaltrials.gov from 1946 to June 21, 2021 and assessed the quality of the studies using the Newcastle–Ottawa Scale. We calculated the pooled OR and the crude rate for suicidal ideation, suicide attempts and completed suicide in patients with epilepsy (PWE).

**Results:**

We screened 2,786 studies and included 88 articles with 1,178,401 PWE and 6,900,657 participants as controls. Search terms included epilepsy and suicide. The pooled rates of suicidal ideation, suicide attempts and completed suicide in PWE were 19.73% (95% CI: 17.00–22.62%), 5.96% (95% CI: 4.82–7.20%), and 0.24% (95% CI: 0.11–0.42%), respectively. Compared to the control group, PWE were at a significantly higher risk of total suicidality (pooled OR, 2.60; 95%: 2.13–3.18), including suicidal ideation (pooled OR, 2.70; 95% CI, 2.21–3.30), suicide attempts (pooled OR, 2.74; 95% CI, 2.08–3.61) and completed suicide (pooled OR, 2.36; 95% CI, 1.45–3.83). Subgroup analyses showed significant differences in the subgroups of the measurement of suicidality.

**Conclusion:**

The rate of suicidal ideation, suicide attempts and completed suicide in PWE were about 19.73, 5.96, and 0.24%. And there was an increased risk of suicidality in PWE especially temporal lobe epilepsy and drug-resistant epilepsy. Clinicians need to be aware of this risk in PWE with early identification and prevention at the time of diagnosis.

Protocol Registration: PROSPERO CRD42021278220.

## 1. Introduction

Epilepsy is a common chronic neurological disorder that affects approximately 50 million people worldwide (1), and nearly a quarter of them have emotional problems (2). Social distress, vulnerability, and reduced quality of life and stigma contributed to the overall profound psychosocial burden in patients with epilepsy (PWE) (2). The burden of suicide constitutes a serious public health issue worldwide. Approximately 800,000 people die from suicide annually according to a global estimate from the World Health Organization (WHO) (3). Suicide is also a cause of premature mortality in PWE. The estimated lifetime prevalence of suicidal attempts among PWE ranged from 3.3 to 14.3%, whereas approximately 38% of PWE had suicidal ideation (4, 5).

In recent years, an increasing number of studies have focused on suicidality in PWE. Suicidality includes suicidal ideation, suicide attempts, and completed suicides (6). Most studies reported one or two components of suicidality in PWE (7–9). A recent meta-analysis only reported the comparison of suicide attempts among PWE and controls and did not report the risk of suicidal ideation and completed suicide (10). The result of the risk of completed suicide in PWE is inconsistent (11–13). It has been reported that 11.5% of all deaths in PWE are a result of suicide, which is 2.06–4.6 times higher than the rate of suicide in the general population (2, 14, 15). However, several studies found a higher risk of completed suicide in PWE than in controls, but the difference was not statistically significant (12, 13). Therefore, it is necessary to determine whether PWE have a higher risk of suicidality. We performed this systematic review and meta-analysis to determine the association between epilepsy and suicidality, including suicidal ideation, suicide attempts and completed suicide.

## 2. Materials and methods

### 2.1. Search strategy and selection criteria

We performed a systematic review and meta-analysis in accordance with Preferred Reporting Items for Systematic Reviews and Meta-Analyses (PRISMA) principles to investigate the association between epilepsy and suicidality. We searched the online databases of PubMed, Embase, Cochrane Online Library, and Clinicaltrials.gov.^1^ The last search was performed on June 21, 2021. Search terms included epilepsy and suicide. Two reviewers independently reviewed the titles and abstracts for any potentially relevant articles.

The two reviewers independently assessed the eligibility of potentially relevant studies according to the predesigned inclusion and exclusion criteria. Disputes about relevance were resolved by consensus among the investigators. The inclusion criteria were as follows.

Study design: English language articles; observational or controlled studies; population: study population identified with a clinical diagnosis of epilepsy with any age; outcomes: diagnosis of suicidality through medical records, interview, or questionnaire; suicidality studied in relation to epilepsy; and outcome measures reported. Animal studies, meta-analyses, reviews, case reports and conference abstracts without full articles to evaluate the quality of studies were excluded.

### 2.2. Data analysis

The two reviewers extracted data from the included studies using a data extraction form that included (1) general information: the first author, year of publication and country of study origin; (2) study characteristics: study design, number of individuals with epilepsy, number of individuals with epilepsy with suicidality, number of healthy controls, number of controls with suicidality, sexes and ages for both the epilepsy and control groups and follow-up; and (3) outcomes: the reviewers separately recorded suicidal ideation, suicide attempts and completed suicide. Suicidal ideation included suicidal risk, suicidal thoughts, suicidal tendencies, and suicidality without a defined attempt or behavior. Suicide attempts included suicidal behavior and suicide plans. In addition, the reviewers recorded the instruments used to measure suicidality and the association [hazard ratio (HR), odds ratio (OR), or risk ratio (RR)] between epilepsy and suicidality. If the patients from those studies were included with the same inclusion criteria in the same period and population, we extracted data from the most complete study if several studies had similar data resources or databases.

Two independent evaluators assessed the quality of the studies using the Newcastle–Ottawa Scale (NOS) from three aspects: the selection of the study groups, the comparability of the groups, and the ascertainment of the outcomes of the studies. The total score was 9; a score ≥ 6 indicated high-quality literature, and a score < 6 indicated low-quality literature.^2^

All statistical analyses were performed using R 4.1.0. We calculated pooled OR in the studies with a control group for suicidal ideation, suicide attempts and completed suicide. We also calculated the crude rates for suicidal ideation, suicide attempts and completed suicide in all included studies with or without control groups. We performed a Shapiro–Wilk normality test to test whether the data fit a normal distribution. If we transformed the rate of suicidality in epilepsy *via* Freeman-Tukey double arcsine transformation, we performed a meta-analysis. The test level α of the effect was set to 0.05. Statistical heterogeneity was evaluated by the I^2^ statistic. The fixed-effects model was used for comparisons with I^2^ < 50%, and the random-effects model was applied for comparisons with I^2^ ≥ 50%. Sensitivity analysis was used to evaluate the stability of the meta-analysis results by omitting every study.

We also performed prespecified analyses to evaluate subgroup differences in the pooled OR and incidence rate by country, age of the population, and measurement of suicidality. We also performed the subgroup analysis to calculate the pooled rate by specific type of epilepsy. We classified the studies into three subgroups according to different types of measurements for suicidality, including diagnostic interviews (such as the Mini International Neuropsychiatric Interview), questionnaires (such as the Beck Depression Inventory and Patient Health Questionnaire-9), and medical records (such as the International Classification of Diseases code). Using meta-regression, we evaluated the effects of year of publication and proportion of women in the study population on the observed associations.

## 3. Results

**Figure 1:**
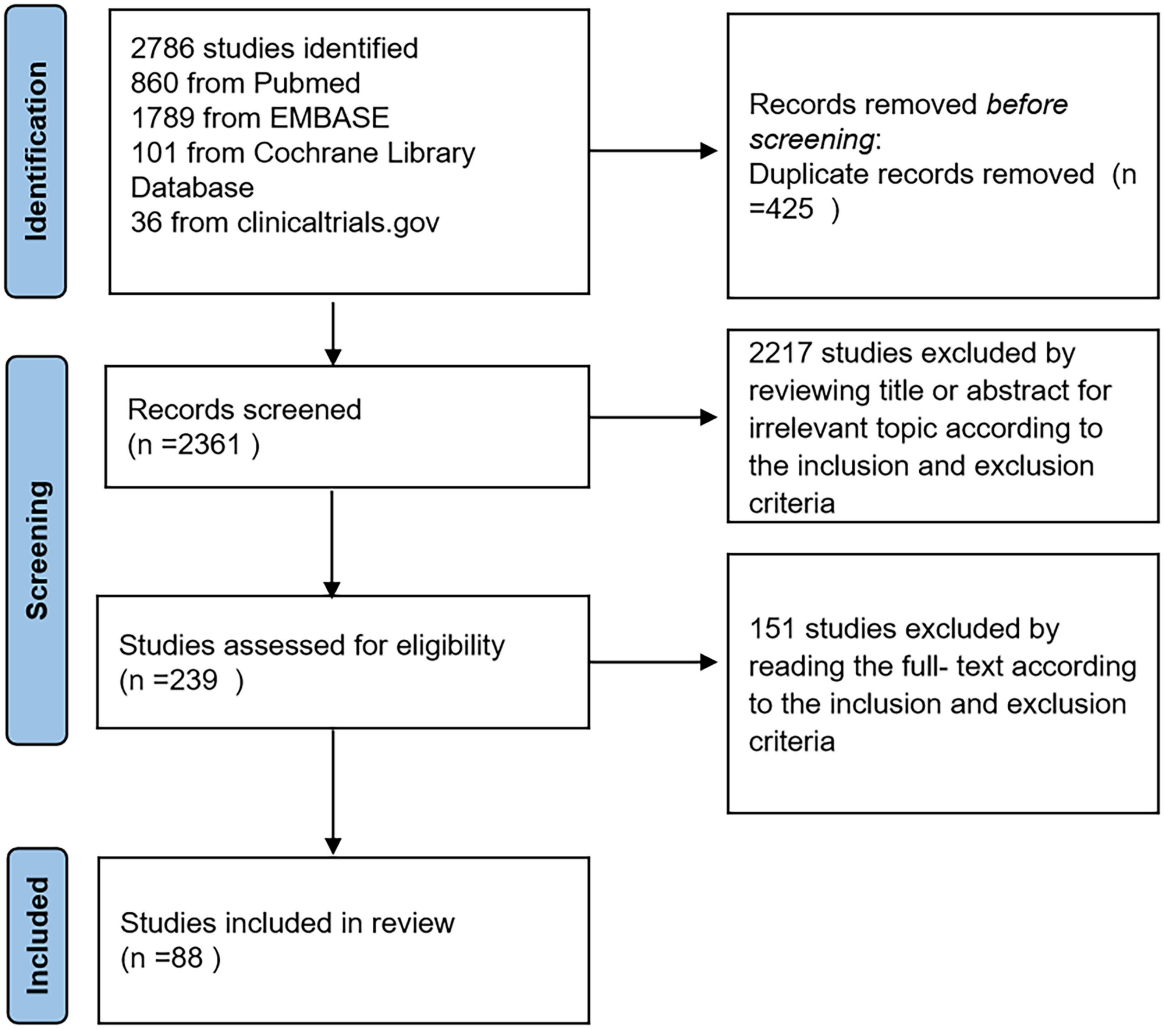
A flow diagram of the article screening process.

**Figure 2:**
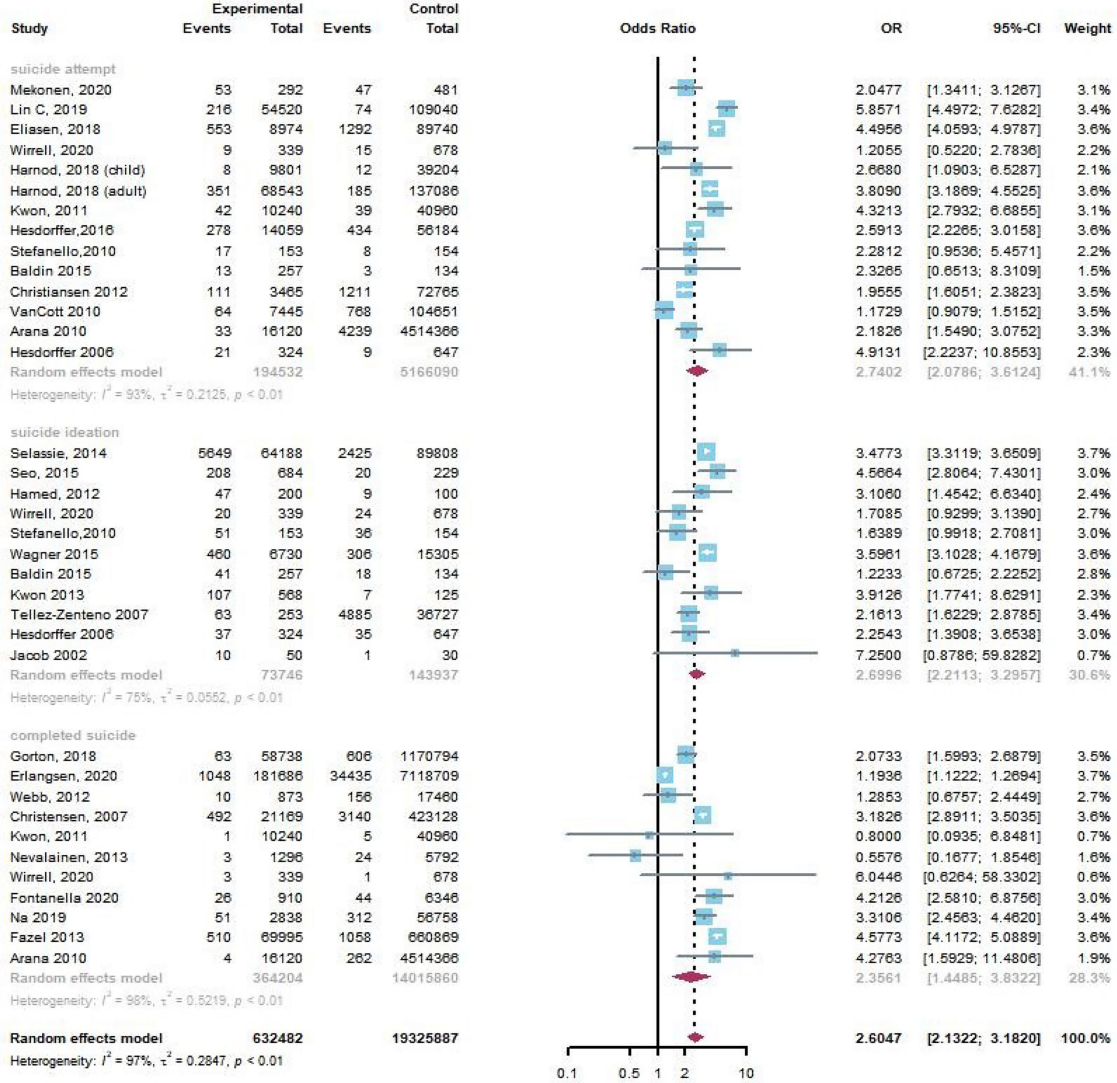
The risk of suicidality between patients with epilepsy and controls.

We screened 2,786 studies and selected 239 studies for full-text review. Following screening, we included 88 articles in our meta-analysis. A flow diagram of the article screening process according to PRISMA is shown in Figure 1 and the PRISMA checklist is shown in Supplementary Table S1. Among a total of 8,079,058 participants, 1,178,401 were PWE, and 6,900,657 participants were controls. There were 35 cohort studies, 42 cross-sectional studies, 10 case–control studies and 1 randomized controlled experiment (RCT) (16). Among studies with a control group, 11 articles reported suicidal ideation, 14 articles reported suicide attempts, and 11 articles reported completed suicide. Sixty-eight articles studied only adults, 10 articles studied only children (<18 years old), and 4 articles studied both adults and children. Other 6 articles did not report the age of population. Six articles addressed newly diagnosed with epilepsy (NDE), 5 articles addressed temporal lobe epilepsy (TLE), and 5 articles addressed drug-resistant epilepsy (DRE). Others did not specify the type of epilepsy. The population in the study of Mula (17) was from Italy Germany and France. We classified this study into a subgroup of developed countries in subgroup analysis because of the larger population in Germany. NOS scores ranged from 5 to 9. The characteristics of the included studies are shown in Supplementary Table S2. PWE were at a higher total risk of suicidality than the general population (pooled OR, 2.60; 95%: 2.13–3.18, *p* < 0.0001, Figure 2).

**Figure 3:**
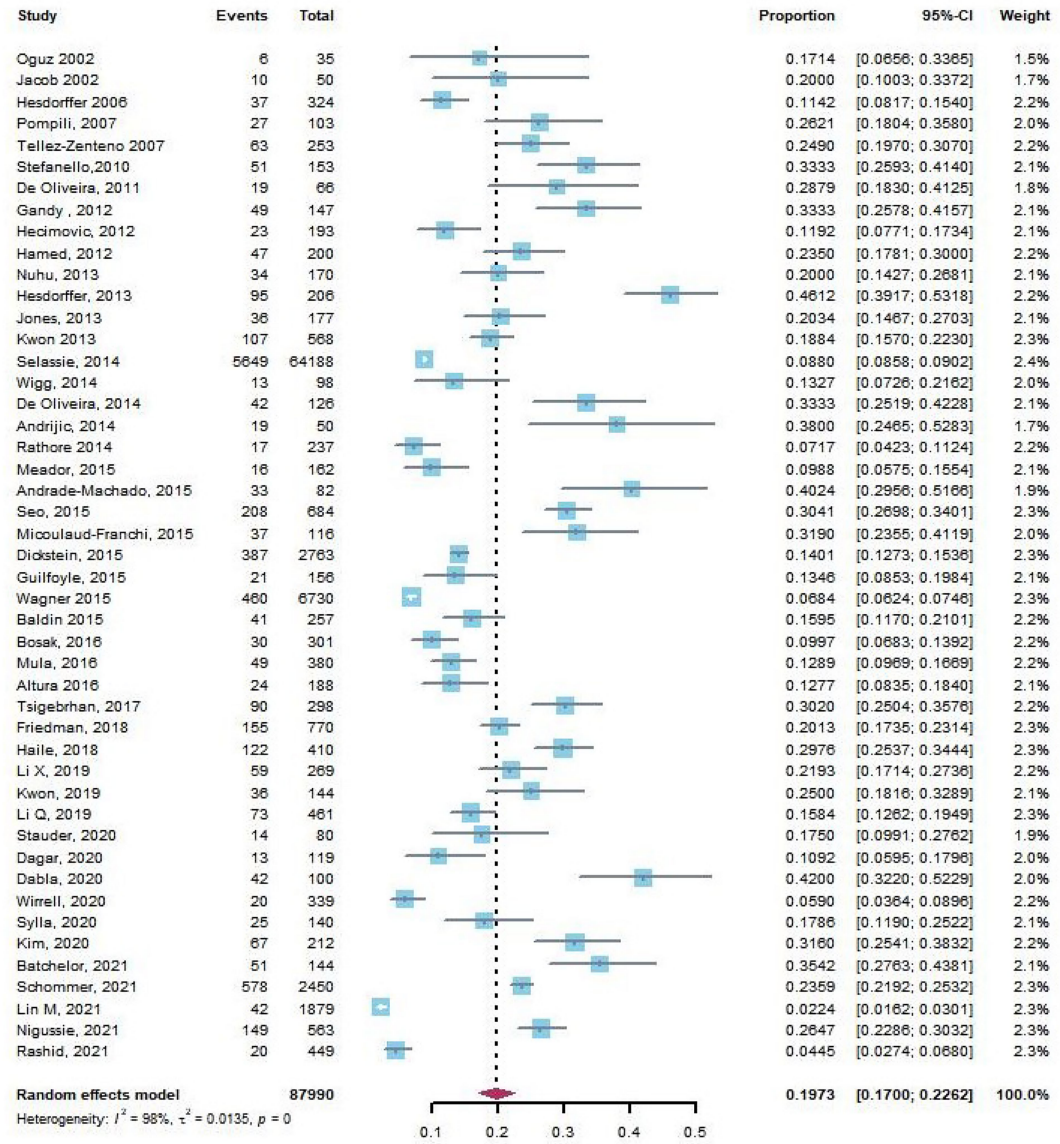
The crude rate of suicidal ideation in patients with epilepsy.

There were 47 articles reporting suicidal ideation in PWE. Based on the pooled data from 11 of 47 articles with a control group, we found that PWE were significantly more likely to experience suicidal ideation than those without epilepsy (pooled OR, 2.70; 95% CI, 2.21–3.30, *p* < 0.0001). The funnel plot was symmetrical, and Egger’s regression test suggested limited publication bias among these studies (*p* = 0.08). We also found that the pooled prevalence rate of suicidal ideation in PWE in 47 studies was 19.73% (95% CI: 17.00–22.62%, Figure 3).

**Figure 4:**
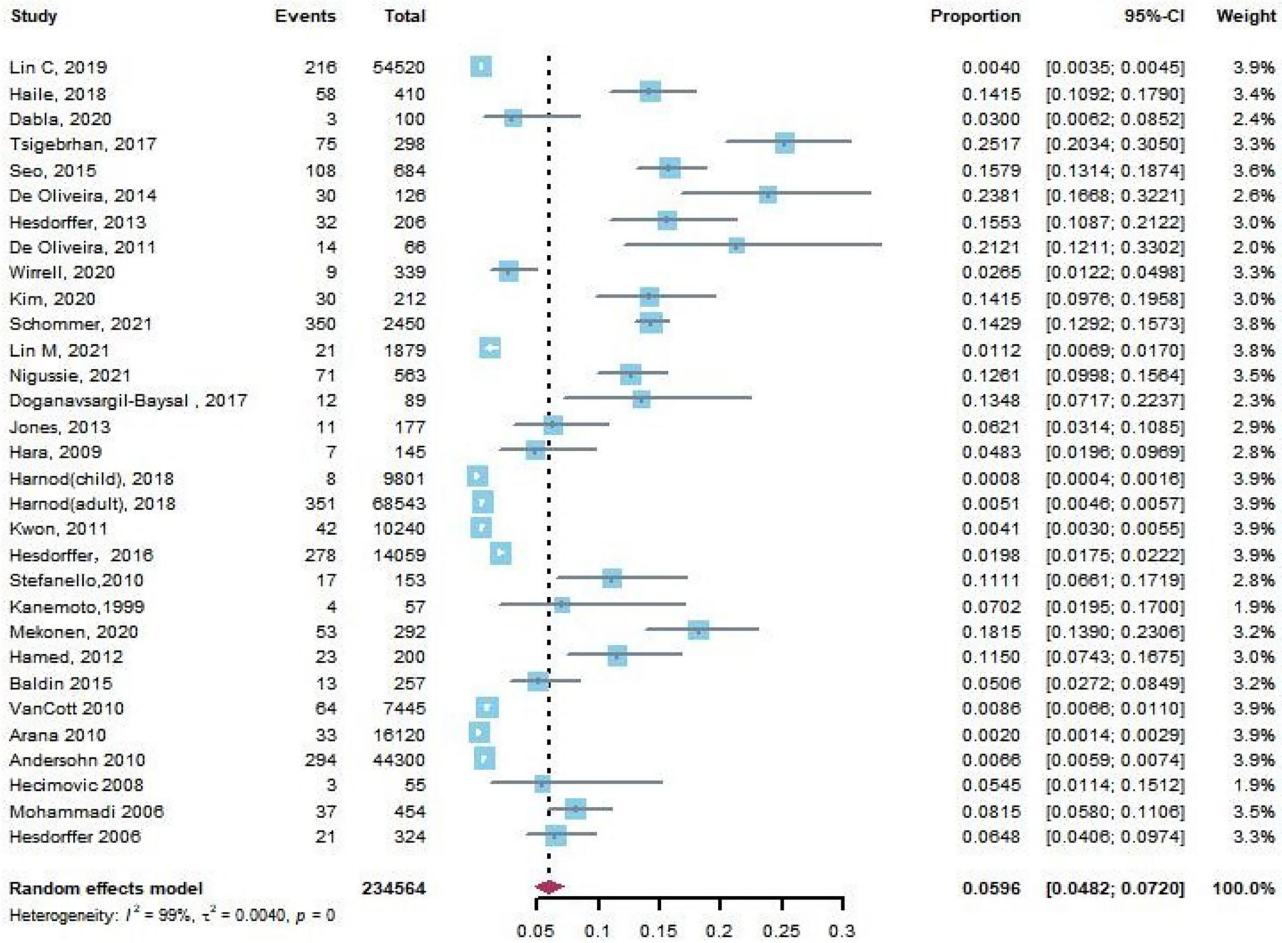
The crude rate of suicide attempts in patients with epilepsy.

Thirty-three articles reported suicide attempts in PWE. Based on the pooled data from 14 of 33 articles with a control group, we found that PWE were significantly more likely to experience suicide attempts than those without epilepsy (pooled OR, 2.74; 95% CI, 2.08–3.61, *p* < 0.0001). The funnel plot was symmetrical, and Egger’s regression test suggested limited publication bias among these studies (*p* = 0.25). Because the study designs of two articles (18, 19) were case–control studies, we excluded them to calculate the pooled prevalence rate of suicide attempts in PWE in 31 studies, and the result was 5.96% (95% CI: 4.82–7.20%, Figure 4).

**Figure 5:**
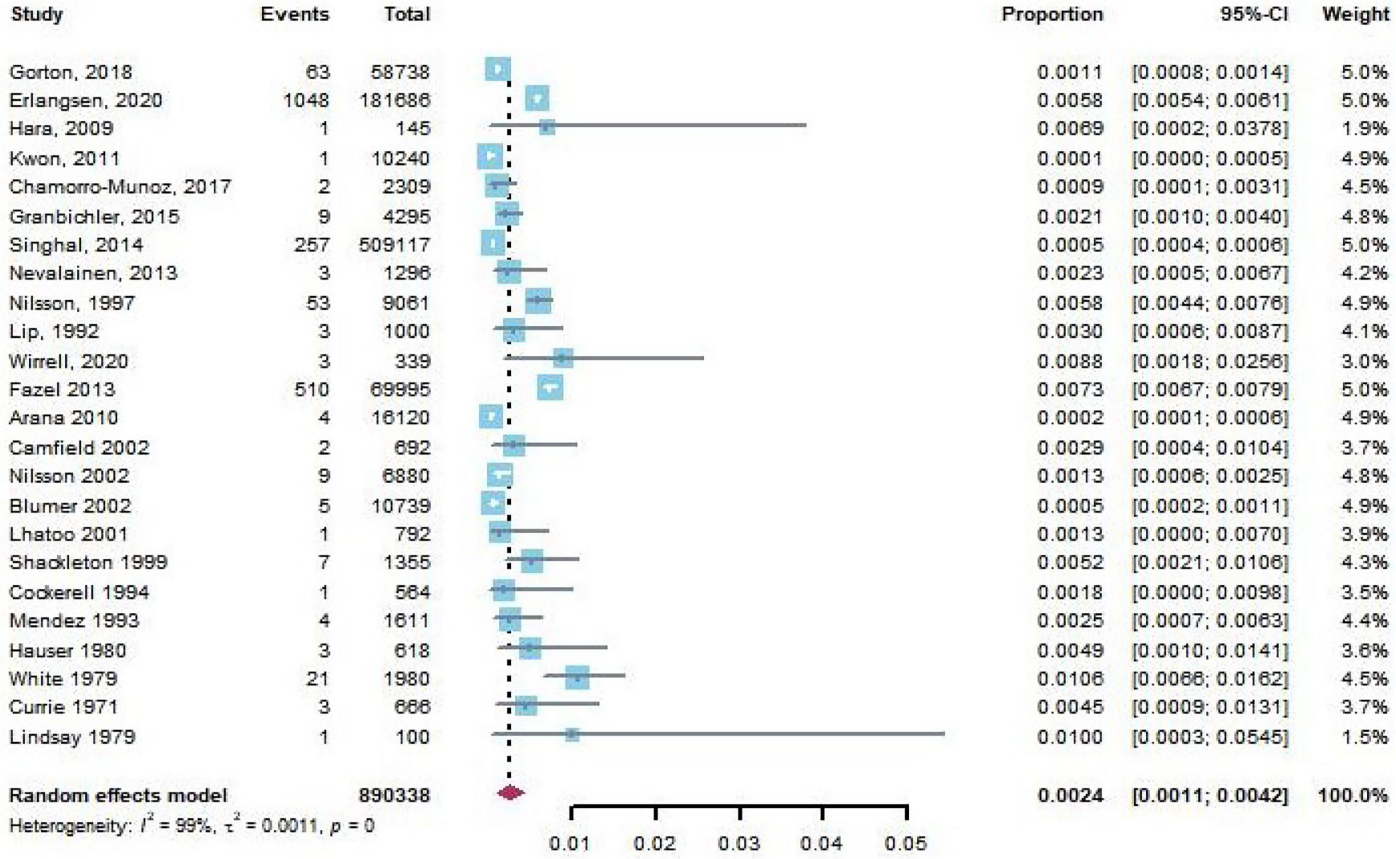
The crude rate of completed suicide in patients with epilepsy.

Twenty-nine articles reported complete suicide in PWE. Based on the pooled data from 11 of 29 articles with a control group, we found that there was significant difference in the risk of completed suicide between the PWE and those without epilepsy (pooled OR, 2.36; 95% CI, 1.45–3.83, *p* = 0.0006). The funnel plot was symmetrical, and Egger’s regression test suggested limited publication bias among these studies (*p* = 0.50). Because the study designs of 5 articles (20–24) were case–control studies, we excluded them to calculate the pooled prevalence rate of completed suicide in PWE in 24 studies, and the result was 0.24% (95% CI: 0.11–0.42%, Figure 5).

The results of prespecified subgroup analyses and meta-regression are shown in Supplementary Table S3. We performed subgroup analyses of the total risk of suicidality, including suicidal ideation, suicide attempts and completed suicide. Subgroup analyses of population about adult and child found no significant difference in suicidality. The rate of suicide attempts in developing countries was significantly higher than that in developed countries. There was no significant difference between different measurements of suicidality in the risk of suicidality. However, the rate of suicidal ideation diagnosed using interviews was higher than that diagnosed using other measurements of suicidality (Interview vs. Questionnaire vs. Medical record: pooled rate 0.22 vs. 0.18 vs. 0.08, *p* < 0.0001). The rate of suicide attempts diagnosed using interviews was similarly higher to those of other measurements of suicidality (Interview vs. Questionnaire vs. Medical record: pooled rate 0.12 vs. 0.09 vs. 0.01, *p* < 0.0001). The diagnosis of completed suicide was identified by medical records. The pooled prevalence rate of suicidal ideation in NDE, TLE and DRE were, respectively, 0.12, 0.27, and 0.29. And the pooled prevalence rate of suicidal attempts in NDE, TLE and DRE were, respectively, 0.02, 0.13, and 0.16.

The meta-regression showed that average age of population (slope 0.03 [0.0022–0.0565], *p* = 0.0342) was a significant moderator that contributed to heterogeneity between the studies in the risk of suicidality. The year of publication and the percentage of females may not contribute to the heterogeneity between studies (*p* > 0.05). The sensitivity analyses showed that the results of the meta-analysis were stable (Supplementary Figures S1–S4). The publication bias of the risk of suicidality were not significant and the funnel plots were shown in Supplementary Figures S5–S7.

## 4. Discussion

This systematic review and meta-analysis found that PWE have a significantly greater risk of suicidality (2.60 times), including suicidal ideation (2.70 times), suicide attempts (2.74 times) and completed suicide (2.36 times). The rates of suicidal ideation, suicide attempts and completed suicide in PWE were 19.73, 5.96, and 0.24%, respectively. This result is consistent with previous studies, which concluded that there was a clear relationship between epilepsy and suicide attempts and completed suicide (10, 25). Previous studies have focused more on suicide attempts and completed suicide. In this meta-analysis, we also found that the risk of suicidal ideation in epilepsy was significantly higher than that in the general population (2)^,^ which was not a consensus previously.

In the subgroup analysis, we found that the different diagnoses may not have influence on the relative risk of suicidality but affected the rate of suicidality, including suicidal ideation and attempts. We found that the rate of suicidality diagnosed by interviews was higher than that diagnosed by questionnaires and medical records. A lack of enough knowledge to seek medical attention in time, a lack of diagnosis from doctors or a lack of medical resources may cause the lower rate of suicidality identified by medical records. This result suggested that using medical records to diagnose suicide may underestimate suicidal rates. Analyses in different types of epilepsy found that there was a higher rate of suicide in DRE and TLE. The suicide rates reported for people with TLE were 6–25%, compared with 1.4–6.9% in the general population (20). Patients with TLE and DRE have a higher frequency of seizures, which is also associated with suicide (15).

Subgroup analyses found that the rate of suicidal ideation in developing countries was significantly higher than that in developed countries, consistent with the findings of the WHO World Mental Health Surveys (26). The WHO reported that 78% of deaths from suicide worldwide occurred in low-income countries (3). At the same time, we found no significant differences in the risk of suicide between adults and children with epilepsy. However, meta- regression showed that the increased average age of the population may be associated with a higher risk of suicidality. The global suicide rate was lowest in patients younger than 15 years old and steadily increased thereafter until the age of 70 (3). Meta-regression showed that the percentage of females may not affect the results of the meta-analysis. On the contrary, other meta-analysis showed that the percentage of females contributed to heterogeneity between the studies (10). The researcher considered male PWE may attempt suicide by more lethal methods (27). However, the prevalence of major depressive disorder in epilepsy is higher in females than that in males (28). It still need more studies to explore the relationship between gender and suicide.

The relationship between epilepsy and suicidality is complex, and epilepsy is a frequently misunderstood and highly stigmatized condition. Epilepsy affects relationships with family and friends, employment, school, and leisure activities and results in social and economic consequences (2, 29). It was reported that psychiatric risk factors, type of epilepsy syndrome and exposure to certain antiseizure medications (ASMs) have been associated with an increased risk of suicide in PWE (30, 31). However, the results about the increased risk of suicide in patients taking ASMs are contradictory (32), with some studies observing an increased suicidality risk (33, 34) and others reporting no increased risk (35, 36). PWE may have a high magnitude of comorbid psychiatric illness (37). Many previous studies have proven that the presence of depression is highly associated with suicidal ideation (21). The possible reason for suicide in PWE might be the direct effect of depression, which can make people feel hopeless and worthless. In addition to psychiatric comorbidity, other common comorbidity in PWE such as traumatic brain injury (38), neurodevelopmental disorders (Autism-spectrum disorder) (39), stroke (40), etc. may also be associated with the increased risk of suicidality.

The mechanism of suicidal behavior in PWE is still uncertain. That may suggest the possible common neurobiological pathogenic mechanisms in suicidality and epilepsy including disturbances of several neurotransmitters and the hypothalamic pituitary adrenal axis (41). Further exploration of depression in PWE will help us to better understand the neurobiology of suicidality and epilepsy (42). Based on the high rate of suicidality among PWE, we should focus on the prevention of suicidality. Countries and communities may lower suicide rates through primary and secondary prevention (43), such as improving seizure control and patient education (44).

The present study had several limitations. First, our included studies had obvious heterogeneity, even after subgroup analysis and meta-regression. This may be due to the different designs, sample sizes, age, sex, duration of epilepsy, comorbid substance use, comorbid depression and anxiety etc. Due to obvious heterogeneity, we cannot specify the pooled prevalence in the meta analysis as the lifetime prevalence or 1-year prevalence. Second, most of the included studies were retrospective, which might have limited the power of our analysis to measure significant differences in outcomes. Similarly, recall bias cannot be excluded. We only searched for articles in English with limited search terms, “epilepsy,” and “suicide”; therefore, several studies may be ignored. Third, the diagnostic tools for assessing suicidality and populations differed among the studies, which may contribute to the heterogeneity. Fourth, there are few articles about specific types of epilepsy in children, which need to further explore in future studies.

This systematic review and meta-analysis found that PWE are at an increased risk of suicidality, including suicidal ideation, suicide attempts and completed suicide. The rate of suicidal ideation, suicide attempts and completed suicide in PWE were about 19.73, 5.96, and 0.24%. In view of the high risk of suicidality in PWE, it is important for clinicians to pay more attention and provide essential screening for early identification and prevention at the time of diagnosis. For especially TLE and DRE, it is necessary to screen by Mini International Neuropsychiatric Interview which can reduce the omission diagnostic rate.

## Data availability statement

The original contributions presented in the study are included in the article/supplementary material, further inquiries can be directed to the corresponding author/s.

## Author contributions

HW and YZ was responsible for the search, data acquisition and interpretation, and drafting the manuscript. HW performed the meta-analysis and generated the figures. YZ was responsible for the study conception and drafting the tables. GT, DC, and YF was responsible for study design and checking the data. LL revised the manuscript and provided the study funding. All coauthors reviewed the final version. All authors had full access to all the data in the study and final responsibility for the decision to submit for publication.

## Funding

This work was supported by Health Commission of Sichuan Province (grant number 20ZD005) and Science & Technology Department of Sichuan Province (grant number 2021YFS0174).

## Conflict of interest

The authors declare that the research was conducted in the absence of any commercial or financial relationships that could be construed as a potential conflict of interest.

## Publisher’s note

All claims expressed in this article are solely those of the authors and do not necessarily represent those of their affiliated organizations, or those of the publisher, the editors and the reviewers. Any product that may be evaluated in this article, or claim that may be made by its manufacturer, is not guaranteed or endorsed by the publisher.

## Footnotes

^1^ https://clinicaltrials.gov/

^2^ http://www.ohri.ca/programs/clinical_epidemiology/oxford.asp
