# Supplementary material for: Suicidality and epilepsy: A systematic review and meta-analysis
Source: Front Psychiatry. 2023 Mar 29;14:1097516. doi: 10.3389/fpsyt.2023.1097516 (PMC10090680; doi:10.3389/fpsyt.2023.1097516)
Supplement: Supplementary file 1 [file Data_Sheet_1.docx]

Supplementary Material

## Supplementary Figures

**
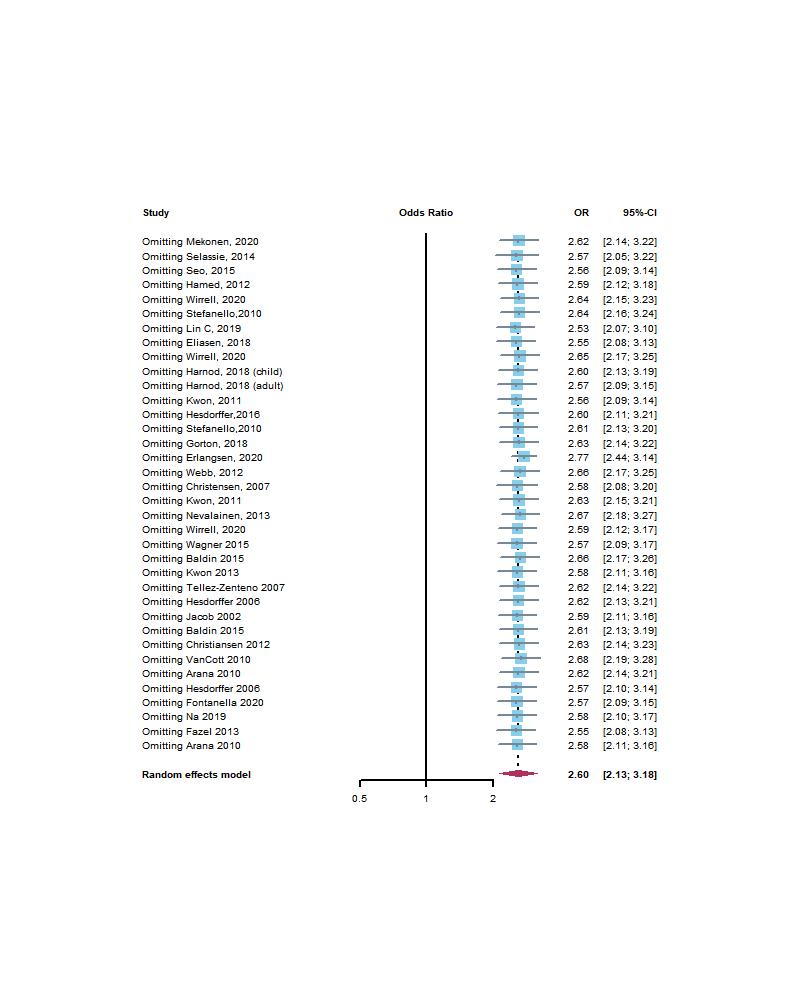
**

**Figure S1. The sensitivity analysis of the risk of suicidality**

**
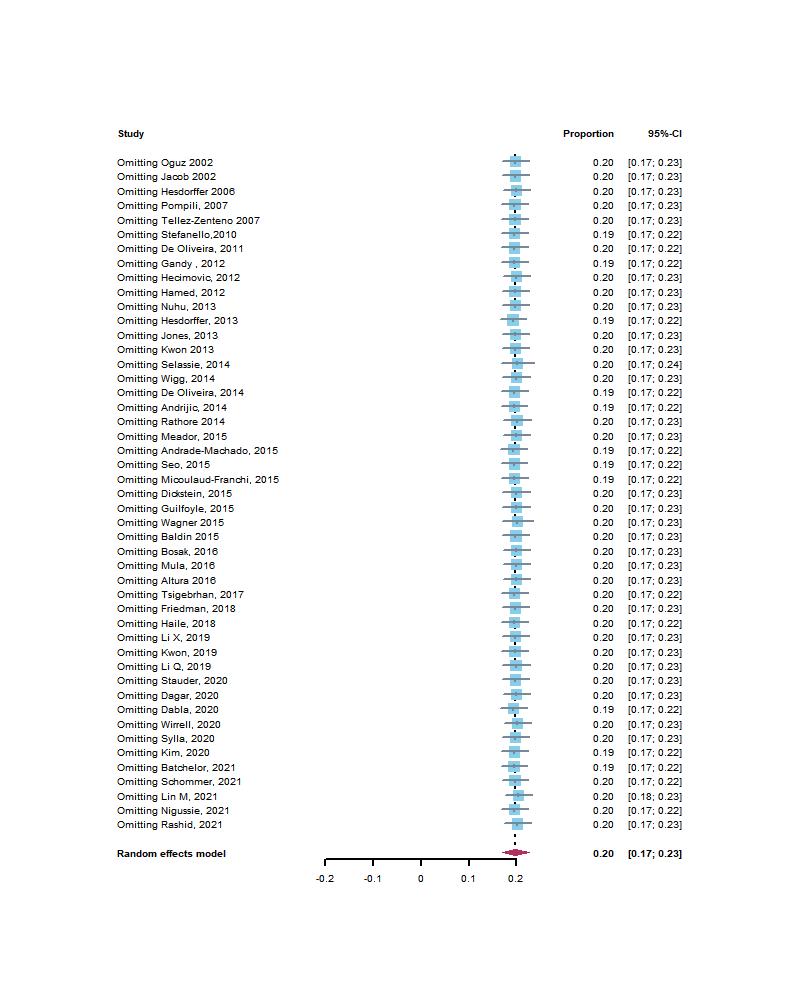
Figure S2. The sensitivity analysis of the rate of suicide ideation**

**
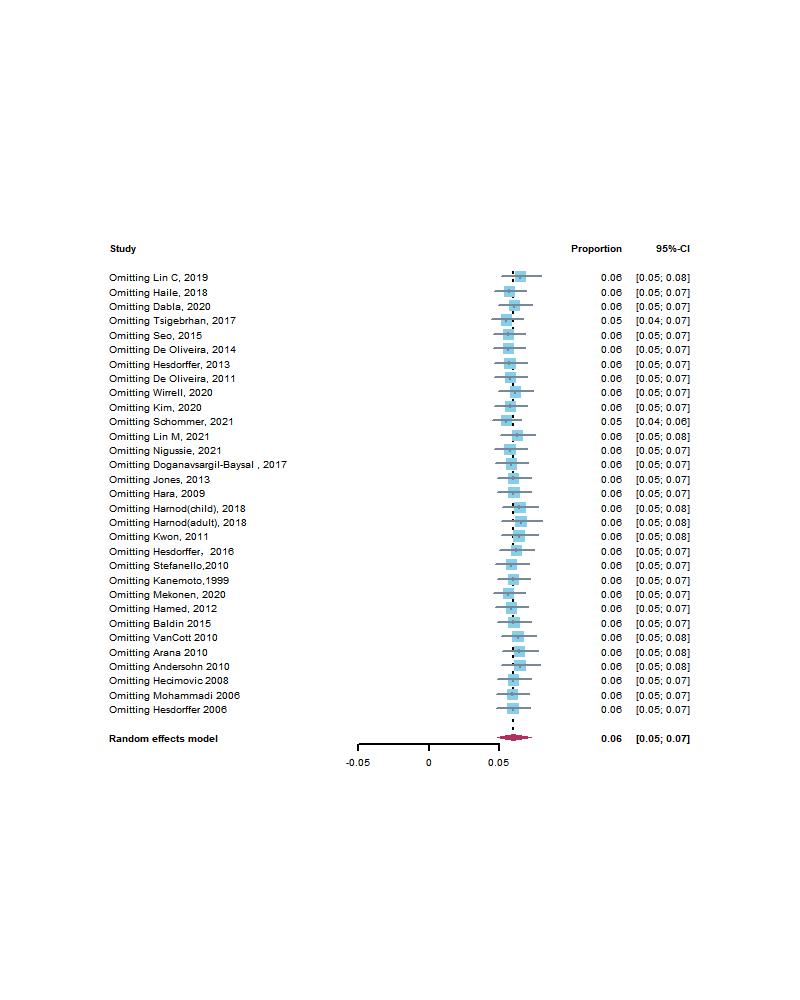
**

**Figure S3. The sensitivity analysis of the rate of suicide attempt**

**
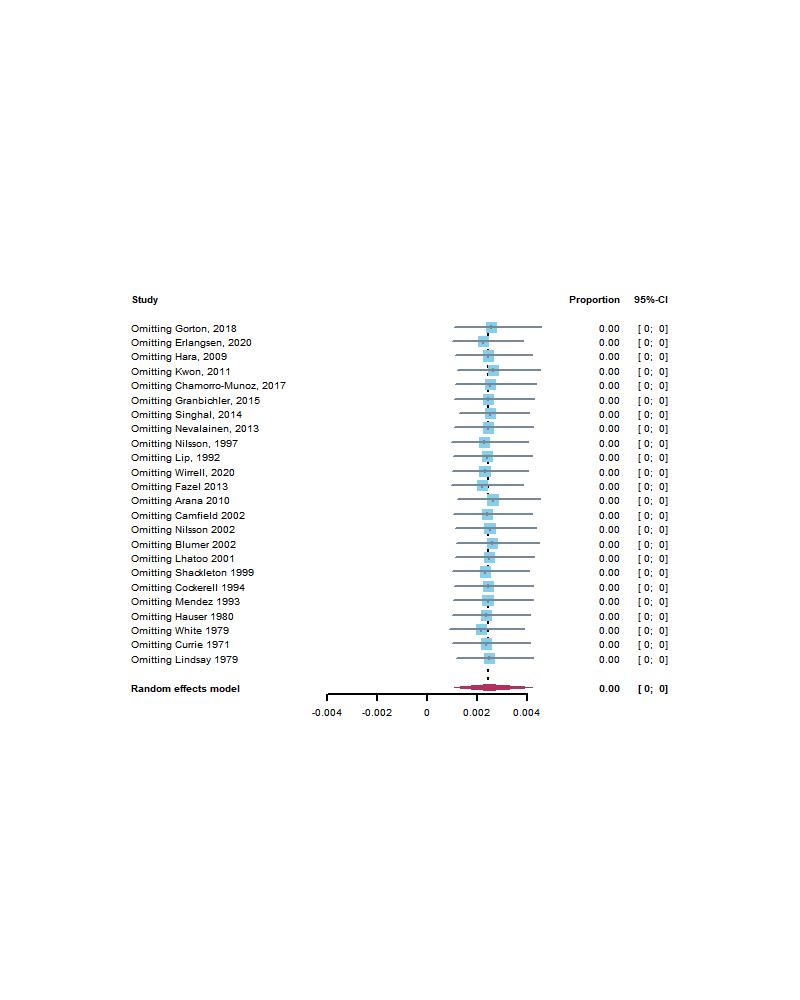
Figure S4. The sensitivity analysis of the rate of completed suicide**


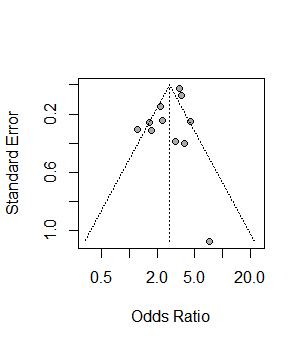


**Figure S5. Funnel plot for evaluation of publication bias in risk of suicide ideation**


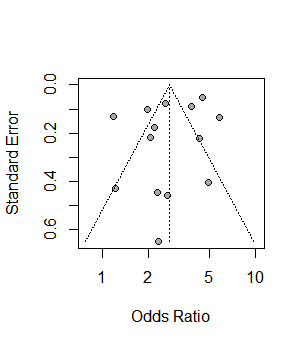


**Figure S6. Funnel plot for evaluation of publication bias in risk of suicide attempt**


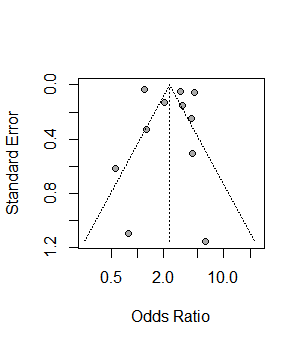


**Figure S7. Funnel plot for evaluation of publication bias in risk of completed suicide**

## Supplementary Tables

**1.2.1 Table S1 PRISMA checklist**

| **Section and Topic** | **Item #** | **Checklist item** | **Location where item is reported** |
| --- | --- | --- | --- |
| **TITLE** | | |  |
| Title | 1 | Identify the report as a systematic review. | Title |
| **ABSTRACT** | | |  |
| Abstract | 2 | See the PRISMA 2020 for Abstracts checklist. | We have checked it in PRISMA- Abstracts checklist. |
| **INTRODUCTION** | | |  |
| Rationale | 3 | Describe the rationale for the review in the context of existing knowledge. | Yes |
| Objectives | 4 | Provide an explicit statement of the objective(s) or question(s) the review addresses. | Yes |
| **METHODS** | | |  |
| Eligibility criteria | 5 | Specify the inclusion and exclusion criteria for the review and how studies were grouped for the syntheses. | Yes |
| Information sources | 6 | Specify all databases, registers, websites, organisations, reference lists and other sources searched or consulted to identify studies. Specify the date when each source was last searched or consulted. | Yes |
| Search strategy | 7 | Present the full search strategies for all databases, registers and websites, including any filters and limits used. | Yes |
| Selection process | 8 | Specify the methods used to decide whether a study met the inclusion criteria of the review, including how many reviewers screened each record and each report retrieved, whether they worked independently, and if applicable, details of automation tools used in the process. | Yes |
| Data collection process | 9 | Specify the methods used to collect data from reports, including how many reviewers collected data from each report, whether they worked independently, any processes for obtaining or confirming data from study investigators, and if applicable, details of automation tools used in the process. | Yes |
| Data items | 10a | List and define all outcomes for which data were sought. Specify whether all results that were compatible with each outcome domain in each study were sought (e.g. for all measures, time points, analyses), and if not, the methods used to decide which results to collect. | Yes |
|  | 10b | List and define all other variables for which data were sought (e.g. participant and intervention characteristics, funding sources). Describe any assumptions made about any missing or unclear information. | Yes |
| Study risk of bias assessment | 11 | Specify the methods used to assess risk of bias in the included studies, including details of the tool(s) used, how many reviewers assessed each study and whether they worked independently, and if applicable, details of automation tools used in the process. | Yes |
| Effect measures | 12 | Specify for each outcome the effect measure(s) (e.g. risk ratio, mean difference) used in the synthesis or presentation of results. | Yes |
| Synthesis methods | 13a | Describe the processes used to decide which studies were eligible for each synthesis (e.g. tabulating the study intervention characteristics and comparing against the planned groups for each synthesis (item #5)). | Yes |
|  | 13b | Describe any methods required to prepare the data for presentation or synthesis, such as handling of missing summary statistics, or data conversions. | Yes |
|  | 13c | Describe any methods used to tabulate or visually display results of individual studies and syntheses. | Yes |
|  | 13d | Describe any methods used to synthesize results and provide a rationale for the choice(s). If meta-analysis was performed, describe the model(s), method(s) to identify the presence and extent of statistical heterogeneity, and software package(s) used. | Yes |
|  | 13e | Describe any methods used to explore possible causes of heterogeneity among study results (e.g. subgroup analysis, meta-regression). | Yes |
|  | 13f | Describe any sensitivity analyses conducted to assess robustness of the synthesized results. | Yes |
| Reporting bias assessment | 14 | Describe any methods used to assess risk of bias due to missing results in a synthesis (arising from reporting biases). | Yes |
| Certainty assessment | 15 | Describe any methods used to assess certainty (or confidence) in the body of evidence for an outcome. | Yes |
| **RESULTS** | | |  |
| Study selection | 16a | Describe the results of the search and selection process, from the number of records identified in the search to the number of studies included in the review, ideally using a flow diagram. | Yes |
|  | 16b | Cite studies that might appear to meet the inclusion criteria, but which were excluded, and explain why they were excluded. | Yes, it was presented in supplement material. |
| Study characteristics | 17 | Cite each included study and present its characteristics. | Yes |
| Risk of bias in studies | 18 | Present assessments of risk of bias for each included study. | Yes,supplement material |
| Results of individual studies | 19 | For all outcomes, present, for each study: (a) summary statistics for each group (where appropriate) and (b) an effect estimate and its precision (e.g. confidence/credible interval), ideally using structured tables or plots. | Yes,supplement material |
| Results of syntheses | 20a | For each synthesis, briefly summarise the characteristics and risk of bias among contributing studies. | Yes |
|  | 20b | Present results of all statistical syntheses conducted. If meta-analysis was done, present for each the summary estimate and its precision (e.g. confidence/credible interval) and measures of statistical heterogeneity. If comparing groups, describe the direction of the effect. | Yes |
|  | 20c | Present results of all investigations of possible causes of heterogeneity among study results. | Yes |
|  | 20d | Present results of all sensitivity analyses conducted to assess the robustness of the synthesized results. | Yes |
| Reporting biases | 21 | Present assessments of risk of bias due to missing results (arising from reporting biases) for each synthesis assessed. | Yes |
| Certainty of evidence | 22 | Present assessments of certainty (or confidence) in the body of evidence for each outcome assessed. | Yes |
| **DISCUSSION** | | |  |
| Discussion | 23a | Provide a general interpretation of the results in the context of other evidence. | Yes |
|  | 23b | Discuss any limitations of the evidence included in the review. | Yes |
|  | 23c | Discuss any limitations of the review processes used. | Yes |
|  | 23d | Discuss implications of the results for practice, policy, and future research. | Yes |
| **OTHER INFORMATION** | | |  |
| Registration and protocol | 24a | Provide registration information for the review, including register name and registration number, or state that the review was not registered. | PROSPERO Haijiao Wang, Yu Zhang, CRD42021278220 |
|  | 24b | Indicate where the review protocol can be accessed, or state that a protocol was not prepared. | PROSPERO |
|  | 24c | Describe and explain any amendments to information provided at registration or in the protocol. | No |
| Support | 25 | Describe sources of financial or non-financial support for the review, and the role of the funders or sponsors in the review. | Yes |
| Competing interests | 26 | Declare any competing interests of review authors. | Yes |
| Availability of data, code and other materials | 27 | Report which of the following are publicly available and where they can be found: template data collection forms; data extracted from included studies; data used for all analyses; analytic code; any other materials used in the review. | Yes |

**1.2.2 Table S2. Characteristics of included studies.**

| Author | Country | Study design | PWE group | | | | | | Control group | | | | | Follow up, year | Unadjusted RR (95% CI) | Adjusted RR (95% CI) | Covariates adjusted factor | NOS |
| --- | --- | --- | --- | --- | --- | --- | --- | --- | --- | --- | --- | --- | --- | --- | --- | --- | --- | --- |
|  |  |  | N of PWE | Population | Suicide in PWE | Age, year | Female, % | Diagnosis of suicidaty | N of Control | Population | Suicide in Control | Age, year | Female, % |  |  |  |  |  |
| Currie,1971 | UK | Cohort study | 666 | TLE | 3 CS | 28 | 52.5 | Medical records | NA | NA | NA | NA | NA | 7 | NA | NA | NA | 5 |
| Lindsay,1979 | UK | Cohort study | 100 | TLE | 1 CS | 19-39 | 37 | Medical records | NA | NA | NA | NA | NA | 13 | NA | NA | NA | 5 |
| White,1979 | UK | Cohort study | 1980 | Epilepsy | 21 CS | NR | NR | ICD | NA | NA | NA | NA | NA | 26 | NA | NA | NA | 6 |
| Hauser,1980 | USA | Cohort study | 618 | Epilepsy | 3 CS | 1-19 | NR | Medical records | NA | NA | NA | NA | NA | 39 | NA | NA | NA | 5 |
| Lip, 1992 | UK | Cohort study | 1000 | Epilepsy | 3 CS | 32(22-68) | 54.8 | Hospital case-notes | NA | NA | NA | NA | NA | 2.3±1.5 | NA | NA | NA | 6 |
| Mendez,1993 | USA | Cohort study | 1611 | Epilepsy | 4 CS | NR | NR | Medical records | NA | NA | NA | NA | NA | 5 | NA | NA | NA | 6 |
| [Cockerell,1994](https://pubmed.ncbi.nlm.nih.gov/?term=Cockerell+OC&cauthor_id=7934347) | UK | Cohort study | 564 | Epilepsy | 1 CS | NR | NR | ICD-9 | NA | NA | NA | NA | NA | 8 | NA | NA | NA | 6 |
| Nilsson, 1997 | Sweden | Cohort study | 9061 | Epilepsy | 53 CS | 54.3(15-97) | 40.7 | ICD-8 or ICD-9 | NA | NA | NA | NA | NA | NA | NA | 3.5(2.6-4.6) | NR | 6 |
| Kanemoto,1999 | Japan | cross-sectional study | 57 | TLE with postictal psychosis | 4 SA | 37.7±14 | 43.3 | NR | NA | NA | NA | NA | NA | NA | NA | NA | NA | 5 |
| Shackleton,1999 | Netherlands | Cohort study | 1355 | Epilepsy | 7 CS | 19(0.5-70) | 45 | Medical records | NA | NA | NA | NA | NA | 28 | NA | NA | NA | 6 |
| Lhatoo,2001 | UK | Cohort study | 792 | Epilepsy | 1 CS | NR | NR | ICD-10 | NA | NA | NA | NA | NA | 11.8 | NA | NA | NA | 6 |
| Oguz,2002 | Turkey | Cohort study | 35 | Epilepsy | 6 SI | 12.9 ± 2.52 | NR | STAI and CDI | 35 | Healthy children | NR | NA | NA | NA | NA | NA | NA | 8 |
| Camfield,2002 | Canada | Cohort study | 692 | Epilepsy | 2 CS | 28d-16y | 50 | Death certificates | NA | NA | NA | NA | NA | 8 | NA | NA | NA | 7 |
| Nilsson,2002 | Sweden | Cohort study | 6880 | Epilepsy | 9 CS | 17-75 | NR | ICD-9 or ICD-10 | NA | NA | NA | NA | NA | 9 | NA | NA | NA | 6 |
| Blumer,2002 | usa | Cohort study | 10739 | Epilepsy | 5 CS | NR | NR | Medical records | NA | NA | NA | NA | NA | 12 | NA | NA | NA | 5 |
| Jacob,2002 | India | Cohort study | 50 | Epilepsy | 10 SI | 33.5 ± 9.0 | 50 | HDRS | 30 | Bronchial asthma | 12SI | 36.5 ± 8.8 | 47 | NR | NA | NA | NA | 7 |
| Mohammadi,2006 | Iran | Cross-sectional study | 454 | Epilepsy | 37 SA | >18 | 56.2 | SADS | NA | NA | NA | NA | NA | NA | NA | NA | NA | 6 |
| Hesdorffer,2006 | The Republic of Iceland | Case-Control Study | 324 | NDE | 37 SI, 21 SA | 34.0(19.9–62.5) | 50.6 | Structured telephone interview | 647 | Age-matched controls | 35 SI, 9 SA | 33.5 (19.9–62.2) | 50.7 | NA | NA | SI: 0.5(0.2-1.2)  SA: 3.9(1.4-11.5) | Age, sex, and cumulative alcohol intake up until the onset of depressive symptoms | 7 |
| Christensen, 2007 | Denmark | Case-Control Study | 21169 | Death with suicide | 492 | 52.1±17.7 | 35.4 | ICD-8 or ICD-10 | 423128 | Matched total population | 3140 CS | 52.1 ±17.7 | 32.9 | NA | 3.17 (2.88–3.50) | 1.9(1.71–2.32) | Comorbid psychiatric disease | 9 |
| Pompili, 2007 | Italy | Cross-sectional study | 103 | TLE | 27 SI | 41.6 ± 14.1 | 69.9 | BHS | NA | NA | NA | NA | NA | NA | NA | NA | NA | 6 |
| Tellez-Zenteno,2007 | Canada | Cross-sectional study | 253 | Epilepsy | 63 SI | >15 | NR | CCHS | 36727 | Non- epilepsy | 4885 | >15 | NR | NA | 2.2 (1.4–3.3) | NA | NA | 7 |
| Hecimovic,2008 | Croatia | Cross-sectional study | 55 | Epilepsy | 3 SA | 30.8±13.5 | 60.4 | BDI | NA | NA | NA | NA | NA | NA | NA | NA | NA | 6 |
| Hara, 2009 | UK | Cross-sectional study | 145 | Epilepsy | 7 SA, 1 CS | 34.3 ± 30 | 68.3 | ICD-10 | NA | NA | NA | NA | NA | NA | NA | NA | NA | 7 |
| Stefanello,2010 | Brazil | Cohort study | 153 | Epilepsy | 51 SI, 17 SA | >13 | 45.8 | SRQ-20 | 154 | Non- epilepsy | 36 SI, 8 SA | NR | 44.8 | NA | NR | NA | NA | 9 |
| VanCott,2010 | USA | Case-Control Study | 7445 | NDE in older veterans | 64 SA | >60 | NR | ICD-9 | 104651 | Non- epilepsy | 768 SA | >60 | NR | NA | NA | NA | NA | 6 |
| Arana,2010 | UK | Cohort study | 16120 | Epilepsy | 33 SA,4 CS | 35.3±20.9 | 53.1 | Code from database | 4514366 | Non- epilepsy | 4239 SA, 262 CS | 32±23.1 | 50.4 | 6.2±5.2 | NR | SA:3.34(2.34-4.78)  CS:NA | Age; duration of illness; status with respect to previous use of antiepileptic drugs, lithium, antipsychotic drugs, or antidepressants; presence or absence of a history of alcohol abuse or a mental disorder; and chronic disease score | 9 |
| Andersohn,2010 | German | nested case-control study | 44300 | Epilepsy | 294 SA | 36.2 (14.0) | 51.2 | Medical records | NA | NA | NA | NA | 5.5 | NA | NA | NA | NA | 7 |
| Kwon, 2011 | Canada | Cohort study | 10240 | Epilepsy | 42 SA, 1 CS | 39.0 ± 21.3 | 48.5 | ICD-9 or ICD-10 | 40960 | Matched non- epilepsy | 39 SA, 5 CS | 39.0 ± 21.3 | 48.5 | NA | SA or CS: 4.32(2.79-6.69) | 1.32 (0.81–2.15) | Comorbid psychiatric disease | 8 |
| De Oliveira, 2011 | Brazil | Cross-sectional study | 66 | TLE | 19 SI, 14 SA | 41.8 ± 10.2 | 53 | MINI | NA | NA | NA | NA | NA | NA | NA | NA | NA | 7 |
| Hamed, 2012 | Egypt | Cross-sectional study | 200 | Epilepsy with low income | 47 SI, 23 SA | 30.47± 7.56 | 50 | NR | 100 | Matched healthy subjects | 9 SI | 29.14 ± 6.90 | 50 | NA | NA | NA | NA | 6 |
| Gandy , 2012 | Australia | Cross-sectional study | 147 | Epilepsy | 49 SI | 39.61±14.14 | 59.2 | MINI | NA | NA | NA | NA | NA | NA | NA | NA | NA | 7 |
| Webb, 2012 | UK | Case-Control Study | 873 | Adult suicides | 10 | 17-98 | 24.6 | ICD-10 | 17460 | Matched living control | 156 CS | 17-98 | 24.6 | NA | 1.33 (0.70-2.55) | 1.10 (0.56-2.14) | Clinical depression | 9 |
| Hecimovic, 2012 | USA | Cross-sectional study | 193 | Epilepsy | 23 SI | 38.76±11.99 | 51.8 | BDI -9 | NA | NA | NA | NA | NA | NA | NA | NA | NA | 6 |
| Christiansen,2012 | Denmark | Case-Control Study | 3465 | SA children and youths | 111 | 10-24 | 79 | ICD | 72 765 | Matched controls | 1211 | 10-24 | 79 | NA | NA | NA | NA | 7 |
| Jones, 2013 | USA | Cross-sectional study | 177 | Epilepsy | 36 SI, 11 SA | 5-16 | NR | K-SADS-PL | NA | NA | NA | NA | NA | NA | NA | NA | NA | 6 |
| Nevalainen, 2013 | Finland | Cohort study | 1296 | NDE | 3 CS | 10-17 | NR | ICD-9 or ICD-10 | 5792 | Population-based reference cohort | 24 CS | 10-17 | NA | 14.6 | 0.57 (0.17–1.90) | NR | NR | 9 |
| Nuhu, 2013 | Nigeria | Cross-sectional study | 170 | Epilepsy | 34 SI | 28.7±12.1 | 41.8 | MINI | NA | NA | NA | NA | NA | NA | NA | NA | NA | 6 |
| Hesdorffer, 2013 | USA | Cross-sectional study | 206 | DRE | 95 SI, 32 SA | 41.2±13.2 | 66.5 | CSSRS | NA | NA | NA | NA | NA | NA | NA | NA | NA | 6 |
| Kwon,2013 | Korea | Cross-sectional study | 568 | Epilepsy | 107 SI | 37.9 ± 13.3 | 38.6 | SSI-Beck | 125 | Healthy controls | 7 SI | 36.4 ± 10.9 | 39.2 | NA | NA | NA | NA | 7 |
| Fazel,2013 | Sweden | Cohort study | 69995 | Epilepsy | 510 CS | 34.5(21.0–44.0) | 47.1 | ICD | 660 869 | Matched general population controls | 1058 CS | 38.9 (27.9–46.7) | 47.3 | 9 (5-18) | NA | NA | NA | 9 |
| Wigg, 2014 | Brazil | Cross-sectional study | 98 | Epilepsy | 13 SI | 39.7 ±12.3 | 39.8 | BDI-9 | NA | NA | NA | NA | NA | NA | NA | NA | NA | 6 |
| Singhal, 2014 | UK | Cohort study | 509117 | Epilepsy | 257 CS | >10 | 49 | ICD-10 | NA | Reference cohort‡ | NA | NA | NA | 1 Y | 1.8 (1.6–2.1) | NR | NR | 8 |
| De Oliveira, 2014 | Brazil | Cross-sectional study | 126 | Epilepsy | 42 SI, 30 SA | 39.3 ±10.2 | 54 | MINI-Plus | NA | NA | NA | NA | NA | NA | NA | NA | NA | 6 |
| Andrijić, 2014 | Bosnia and Herzegovina | Cross-sectional study | 50 | Epilepsy | 19 SI | 38.6 ± 2.2 | 50 | BHS | NA | NA | NA | NA | NA | NA | NA | NA | NA | 7 |
| Selassie, 2014 | USA | Cohort study | 64188 | Epilepsy | 5649 SI | 41.6 ± 22.5 | 51.3 | ICD-9 | 89808 | Lower extremity fracture without pathological fractures | 2425 SI | 38.8± 22.7 | 48.3 | NR | NR | 2.95(2.81–3.10) | Age, race, gender, insurance status, and mortality status and number of comorbid conditions. | 9 |
| Rathore,2014 | USA | Cross-sectional study | 237 | Epilepsy | 17 SI | 40.6 ± 14.2 | 68.8 | MINI | NA | NA | NA | NA | NA | NA | NA | NA | NA | 6 |
| Granbichler, 2015 | Austria | Cohort study | 4295 | Epilepsy | 9 CS | 46.3 (18.0–99.4) | 52.6 | ICD-9 or ICD-10 | NA | NA | NA | NA | NA | 6.4 | 4.2 (2.0–8.1) | NR | NR | 9 |
| Meador, 2015 | USA | RCT | 162 | Partial-onset DRE | 16 SI | 34.9 ± 11.6 | 48 | BDI-II-9 | NA | NA | NA | NA | NA | NA | NA | NA | NA | 6 |
| Andrade-Machado, 2015 | Cuba | Cross-sectional study | 82 | Focal DRE | 33 SI | 38.02 ± 11.26 | 37.8 | MINI | NA | NA | NA | NA | NA | NA | NA | NA | NA | 6 |
| Seo, 2015 | Korea | multicenter, cross-sectional study | 684 | Epilepsy | 208 SI, 108 SA | 41.5 ± 12.2 | 42.4 | MINI | 229 | Non- epilepsy | 20 SI | 41.4±12.1 | 42.8 | NA | NR | NR | NR | 8 |
| Micoulaud-Franchi, 2015 | France | Cross-sectional study | 116 | Epilepsy | 37 SI | 40.39±13.83 | 58.6 | MINI | NA | NA | NA | NA | NA | NA | NA | NA | NA | 6 |
| Dickstein, 2015 | USA | Cross-sectional study | 2763 | Epilepsy | 387 SI | 48±17 | 58 | PHQ-9 | NA | NA | NA | NA | NA | NA | NA | NA | NA | 6 |
| Guilfoyle, 2015 | USA | Cohort study | 156 | NDE | 21 SI | 11.9± 2.8 | 50 | CDI-2 | NA | NA | NA | NA | NA | 5.6 ± 3.5m | NA | NA | NA | 6 |
| Wagner,2015 | USA | Case-Control Study | 6730 | Epilepsy | 460 SI | 14.23 (3.4) | 54.5 | ICD-9 | 15 305 | Children with a fracture of the tibia, fibula, or ankle (LEF group) | 306 SI | 13.9 ±2.9 | 40.3 | NA | 4.42 (3.69–5.29) | 2.16 (1.77–2.63) | Adjusted for neurodevelopmental comorbidities, mortality, and demographic variables | 7 |
| Park,2015 | Korea | Case-Control Study | 35638 | Death with suicide | 135 | 18-75 | NR | ICD-10 | NA | NA | NA | NA | NA | 7.3(0.5-19) | NA | NA | NA | 6 |
| Baldin,2015 | USA | Cohort study | 257 | Childhood-onset epilepsy | 41 SI, 13 SA | 22.5 (3.5) | 49 | DIS-IV | 134 | Sibling controls | 18 SI, 3 SA | 23.6 ±5 | 59 | 9 | SI: 1.2 (0.7-2.3)  SA: 2.5(0.7-9.4) | SI: 1.0 (0.4-2.3)  SA: 3.8(0.6-24.7) | Age, sex | 7 |
| Bosak, 2016 | Poland | Cross-sectional study | 301 | Epilepsy | 30 SI | 35.5 ±14.8 | 59.8 | BDI-9 | NA | NA | NA | NA | NA | NA | NA | NA | NA | 6 |
| Hesdorffer,2016 | UK | Cohort study | 14059 | NDE with first SA | 278 Recurrent SA | 36 (10-60） | 48.2 | ICD | 56184 | Non-epilepsy with first SA | 434 Recurrent SA | 36 (10-60） | 48.2 | 0.5 | 1.8 (1.3-2.5) | 1.8 (1.3-2.5) | Age, sex, and psychiatric disorders as a time-varying covariate | 8 |
| Mula, 2016 | Germany,Italy, France | Cross-sectional study | 380 | Epilepsy | 49 SI | 39.4 ± 14.6 | 53.7 | NDDIE | NA | NA | NA | NA | NA | NA | NA | NA | NA | 6 |
| Altura,2016 | USA | Cross-sectional study | 188 | Epilepsy | 24 SI | 40.6 (18.2–78.8) | 51.3 | PHQ-9 | NA | NA | NA | NA | NA | NA | NA | NA | NA | 6 |
| Chamorro-Munoz, 2017 | Spain | Cohort study | 2309 | Epilepsy | 2 CS | 38.5±17.5 | 45.3 | Medical report | NA | NA | NA | NA | NA | 6.9 (0.03–13.5) | 1.2( 0.13-4.33) | NR | NR | 8 |
| Tsigebrhan, 2017 | Ethiopia | Cross-sectional study | 298 | Epilepsy | 90 SI, 75 SA | 33.3±13.7 | 41.3 | CIDI | NA | NA | NA | NA | NA | NA | NA | NA | NA | 6 |
| Doganavsargil-Baysal , 2017 | Turkey | Cohort study | 89 | Epilepsy | 12 SA | 31.44 ± 1.07 | 50.6 | SCID-IV | NA | NA | NA | NA | NA | 0.5 | NA | NA | NA | 6 |
| Harnod(child), 2018 | China | Cohort study | 9801 | Epilepsy | 8 SA | 9.9± 3.45 | 37.1 | ICD-9 | 39204 | Non- epilepsy | 12 SA | 9.89 ± 3.45 | 55.7 | 5.19±3.18 | NR | 2.67(2.36-2.81) | Age, sex, urbanization level, parental occupation, and the comorbidities of depression, anxiety, mental disorders, and sleep disorders | 9 |
| Harnod(adult),2018 | China | Cohort study | 68543 | Epilepsy | 351 SA | 56.4 ± 20.0 | 37.1 | ICD-9 | 137086 | Non- epilepsy | 185 SA | 55.9 ± 19.9 | 37.1 | NA | NR | NR | NR | 9 |
| Friedman, 2018 | USA | Cross-sectional study | 770 | Epilepsy | 155 SI | 42.4 ± 13.0 | 59.6 | PHQ-9 | NA | NA | NA | NA | NA | NA | NA | NA | NA | 6 |
| Gorton, 2018 | UK (England) | Cohort study | 44687 | Epilepsy | 47 CS | 41 (25-60) | 48.6 | ICD-10 | 891429 | Matched comparators without epilepsy | 407 CS | 40 (25-60) | 48.6 | 4.0 (1.4-8.4) | NR | 2.46(1.80-3.36) | Area-level deprivation | 9 |
| Gorton, 2018 | UK (Wales) | Cohort study | 14051 | Epilepsy | 16 CS | 44 (24-62) | 45.1 | ICD-10 | 279365 | Matched comparators without epilepsy | 199 CS | 43 (24-62) | 49 | 6.9 (2.9-10.3) | NR | 1.68(1.00-2.80) | Area-level deprivation | 9 |
| Haile, 2018 | Ethiopia | Cross-sectional study | 410 | Epilepsy | 122 SI, 58 SA | 32.95± 11.87 | 40.2 | CIDI | NA | NA | NA | NA | NA | NA | NA | NA | NA | 7 |
| Eliasen, 2018 | Denmark | Case-control study | 8974 | Danish Poison Information Centre database with poisoning with suicidal intent | 553 | 39.8 ±13.9 | NR | DPIC | 89740 | Matched living control | 1292 SA | 40.3 ±16.5 | NA | NA | NR | 4.5 (4.1–5.0) | Age and gender | 9 |
| Li X, 2019 | China | Cross-sectional study | 269 | Epilepsy | 59 SI | 16-80 | 43.5 | MINI | NA | NA | NA | NA | NA | NA | NA | NA | NA | 7 |
| Kwon, 2019 | Korea | Cross-sectional study | 144 | DRE | 36 SI | 19-68 | 40.3 | MINI | NA | NA | NA | NA | NA | NA | NA | NA | NA | 7 |
| Li Q, 2019 | China | Cross-sectional study | 461 | Epilepsy | 73 SI | 29.68± 11.46 | 53.8 | NDDI-E | NA | NA | NA | NA | NA | NA | NA | NA | NA | 7 |
| Lin C, 2019 | China | Prospective Cohort study | 54520 | NDE | 216 SA | 53.8 ±19.9 | 37.4 | ICD-9 | 109040 | Non- epilepsy | 74 SA | 53.5 ±19.9 | 37.4 | 13 | 7.07(5.43, 9.21) | 5.49(4.13, 7.29) | Age, monthly income, urbanization level, and comorbidity of schizophrenia,depression, alcohol-related illness, anxiety, mental disorders, and insomnia | 9 |
| Na,2019 | Korea | Case-Control Study | 2838 | Death with suicide | 51 | NR | 32.77 | ICD-10 | 56758 | Matched controls | 312 | NR | 32.77 | NA | NA | NA | NA | 8 |
| Dagar, 2020 | USA | Cross-sectional study | 119 | Epilepsy | 13 SI | 15.76 ±2.44 | 54.6 | ASQ | NA | NA | NA | NA | NA | NA | NA | NA | NA | 7 |
| Mekonen, 2020 | Ethiopia | Cross-sectional study | 292 | Epilepsy | 53 SA | 29.2 ± 10.9 | 47.3 | SBQ-R | 481 | Non- epilepsy | 47 SA | 29.2 ± 10.9 | 54.3 | NA | NR | 1.98(1.2–3.3) | Sociodemographic variables, psychosocial variables, clinical variables† | 9 |
| Erlangsen, 2020 | Denmark | Cohort study | 181686 | Epilepsy | 1048 CS | 23.6(10.0-37.0) | 50.1 | ICD-8 or ICD-10 | 7118 709 | Non- epilepsy | 34435 CS | 23.6(10.0-37.0) | 50.1 | 23.6 (10.0-37.0) | NR | 1.7 (1.6-1.8) | Period, sex, age group, living status, region, socioeconomic status, physical comorbidity, psychiatric hospitalization prior to diagnosis of any neurological disorders, and deliberate self-harm prior to diagnosis of any neurological disorders | 9 |
| Stauder,2020 | Bhutan | Cross-sectional study | 80 | Epilepsy | 14 SI | 29.4 (18-56) | 48.8 | PHQ-9 | NA | NA | NA | NA | NA | NA | NA | NA | NA | 7 |
| Dabla, 2020 | India | Cross-sectional study | 100 | Epilepsy | 42 SI, 3 SA | 26.87±8.27 | 30 | CSSRS | NA | NA | NA | NA | NA | NA | NA | NA | NA | 6 |
| Sylla,2020 | Guinea | Cross-sectional study | 140 | Epilepsy | 25 SI | 16-66 | 45.7 | PHQ-9 | NA | NA | NA | NA | NA | NA | NA | NA | NA | 7 |
| Kim, 2020 | Korea | Cross-sectional study | 212 | Epilepsy | 67 SI, 30 SA | 41.1±11.9 | 47.6 | MINI | NA | NA | NA | NA | NA | NA | NA | NA | NA | 7 |
| Wirrell, 2020 | USA | Cohort study | 339 | Epilepsy | 20 SI, 9 SA, 3 CS | 27.4 (18.1-32.2) | 47.2 | CSSRS | 678 | Non- epilepsy | 24 SI, 15 SA, 1 CS | 23.4(18.0-30.5) | NR | NR | SI:1.56 (1.04-2.35)  SA:1.48(0.93-2.37)  CS:NR | NR | NR | 8 |
| Fontanella,2020 | USA | Case-Control Study | 910 | Death with suicide | 26 | 15.7 (2.0) | 27.1 | ICD-9 | 6346 | Matched controls | 44 | NR | NR | NA | NA | NA | NA | 8 |
| Rashid, 2021 | India | Cross-sectional study | 449 | Epilepsy | 20 SI | 28.8 ± 9.34 | 48.8 | MINI | NA | NA | NA | NA | NA | NA | NA | NA | NA | 7 |
| Batchelor,2021 | UK | Cross-sectional study | 144 | Epilepsy | 51 SI | 21.6±2.2 | 61.1 | NDDI-E | NA | NA | NA | NA | NA | NA | NA | NA | NA | 7 |
| Schommer,2021 | UK | Cross-sectional study | 2450 | Epilepsy | 578 SI, 350 SA | 47 (18-88) | 53 | NDDI-E | NA | NA | NA | NA | NA | NA | NA | NA | NA | 7 |
| Lin M,2021 | China | Cross-sectional study | 1879 | Epilepsy | 42 SI, 21SA | 27.55 (9-26) | NR | MINI | NA | NA | NA | NA | NA | NA | NA | NA | NA | 7 |
| Nigussie,2021 | Ethiopia | Cross-sectional study | 563 | Epilepsy | 149 SI, 71 SA | 29 (23-36) | 42.5 | CIDI | NA | NA | NA | NA | NA | NA | NA | NA | NA | 7 |

† Sociodemographic variables: sex, age, marital status, job, and educational Psychosocial variables: social support, family emotional involvement (perceived criticism), depressive symptoms, alcohol abuse, and khat chewing Clinical variables: type of antiepileptic drugs, duration of epilepsy, and seizure frequency; ‡ The reference cohort was constructed of inpatients with a wide range of other, mainly minor, surgical and medical conditions and injuries; NA: not available; NR: not reported; MINI: Mini International Neuropsychiatric Interview; CSSRS: the Columbia Suicide Severity Rating Scale; BHS: Beck Hopelessness Scale; BDI : Beck Depression Inventory; CDI-2: Children's Depression Inventory—Second Edition; CIDI: composite international diagnostic interview; SCID-IV: Structured Clinical Interview for DSM-IV ; DPIC: Danish Poison Information Centre; ASQ: Ask Suicide-Screening Questions; NDDI-E: Neurological Disorders Depression Inventory for Epilepsy; SBQ-R: Suicidal Behaviors Questionnaire-Revised; PHQ-9: Patient Health Questionnaire -9; HAM-D: Hamilton Depression Rating Scale; K-SADS-PL: Kiddie Schedule for Affective Disorders and Schizophrenia-Present and Lifetime Version; SRQ 20:Self-Reporting Questionnaire ;SSCI-8: Stigma Scale for Chronic Illnesses 8-item version ; STAI：State Trait Anxiety Inventory; SSI-Beck: Beck Scale for Suicidal Ideation ; CDI: Children’s Depression Inventory ; HDRS:Hamilton depression rating scale ; CCHS:Canadian Community Health Survey ; SADS:Schedule for Affective Disorders and Schizophrenia; DIS-IV:Diagnostic Interview Survey ; CS: completed suicide; SI: suicide ideation; SA: suicide attempt; ICD:International Classification of Diseases; TLE: Temporal lobe epilepsy; DRE: Drug resistant epilepsy; NDE: Newly diagnosed epilepsy; USA: United States; UK:United kingdom; RCT:Randomized controlled experiment; PWE: patients with epilepsy ; Y:Year ; M:Month; D:Day.

**1.2.3 Table S3. Prespecified Subgroup Analyses and Meta-Regression.**

| **Subgroup analysis** | **Rate of suicide ideation, 95%CI** | **Rate of suicide attempt, 95%CI** | **Rate of completed suicide, 95%CI** | **OR of total suicidality (ideation, attempt and completed), 95%CI** |
| --- | --- | --- | --- | --- |
| **Country** | *P =* 0.3076 | ***P = 0.0348* *** | *-* | *P = 0.3828* |
| Developed country | 0.1814 [0.1499 - 0.2152] (n=25) | 0.0473 [0.0309 - 0.0669] (n=15) | - | 2.5090 [1.9987 - 3.1497] (n=28) |
| Developing country | 0.2195 [0.1535 - 0.2934] (n=22) | 0.0743 [0.0573 - 0.0938] (n=16) | - | 3.0321 [2.1167 - 4.3434] (n=8) |
| **Population** | *P =* 0.0713 | *P = 0.3136* | *P = 0.6403* | *P = 0.6528* |
| Adult | 0.2029 [0.1706 - 0.2373] (n=42) | 0.0632 [0.0507 - 0.0769] (n=27) | 0.0021 [0.0006 - 0.0041] (n=13) | 2.5126 [1.9817 - 3.1857](n=27) |
| Child | 0.1285 [0.0693 - 0.2018] (n=5) | 0.0271 [0.0000 - 0.1097] (n=3) | 0.0029 [0.0010 - 0.0056] (n=3) | 2.8264 [1.7942 - 4.4526] (n=5) |
| **Measurement of suicidality** | ***P < 0.0001**** | ***P < 0.0001**** | *-* | *P = 0.1151* |
| Interview | 0.2208 [0.1525 - 0.2976] (n=20) | 0.1167 [0.0678 - 0.1765] (n=13) | - | 2.5622 [1.7196 - 3.8176] (n=6) |
| Questionnaire | 0.1898 [0.1585 - 0.2232] (n=25) | 0.0945 [0.0521 - 0.1475] (n=7) | - | 1.6712 [1.1219 - 2.4895] (n=7) |
| Medical record | 0.0781 [0.0600 - 0.0984] (n=2) | 0.0058 [0.0034 - 0.0088] (n=9) | - | 2.7394 [2.1291 - 3.5245] (n=22) |
| **Specific type of epilepsy^†^** |  |  |  |  |
| No specific epilepsy | 0.1896 [0.1611 - 0.2197](n=39) | 0.0660 [0.0504 - 0.0834](n=24) | 0.0024 [0.0010 - 0.0042] (n=21) | 2.6481 [2.1199 - 3.3079] (n=30) |
| NDE | 0.1204 [0.0926 - 0.1513] (n=2) | 0.0168 [0.0064 - 0.0318] (n=4) | 0.0023 [0.0003 - 0.0059] (n=1) | 2.3725 [1.3600 - 4.1387] (n=6) |
| TLE | 0.2719 [0.2068 - 0.3422] (n=2) | 0.1348 [0.0272 - 0.2991] (n=2) | 0.0037 [0.0001 - 0.0103] (n=2) | - |
| DRE | 0.2901 [0.1293 - 0.4835] (n=4) | 0.1553 [0.1088 - 0.2082] (n=1) | - | - |
| **Meta regression** |  |  |  |  |
| Year of publication | *P =* 0.8975 | *P =* 0.8259 | *P =* 0.5707 | *P =* 0.1122 |
| Percentage of females | *P =* 0.3842 | *P =* 0.5037 | *P =* 0.6415 | *P =* 0.0805 |
| Average age of population | 0.0293[0.0022 - 0.0565], ***P = 0.0342**** | *P =* 0.4648 | *P =* 0.3216 | *P =* 0.2876 |

* *P* < 0.05. TLE: Temporal lobe epilepsy; DRE: Drug resistant epilepsy; NDE: Newly diagnosed epilepsy; † For each specific type of epilepsy, we only can calculate the pooled rate without subgroup comparison.
